# Climate Change or Urbanization? Impacts on a Traditional Coffee Production System in East Africa over the Last 80 Years

**DOI:** 10.1371/journal.pone.0051815

**Published:** 2013-01-14

**Authors:** Juliana Jaramillo, Mamoudou Setamou, Eric Muchugu, Adenirin Chabi-Olaye, Alvaro Jaramillo, Joseph Mukabana, Johnson Maina, Simon Gathara, Christian Borgemeister

**Affiliations:** 1 Institute of Plant Diseases and Plant Protection, University of Hannover, Hannover, Germany; 2 International Center of Insect Physiology and Ecology (icipe), Nairobi, Kenya; 3 Centro Nacional de Investigaciones de Café – Cenicafé, Manizales, Colombia; 4 Texas A&M University-Kingsville Citrus Center, Weslaco, Texas, United States of America; 5 Kenya Meteorological Department, Nairobi, Kenya; New York State Museum, United States of America

## Abstract

Global environmental changes (GEC) such as climate change (CC) and climate variability have serious impacts in the tropics, particularly in Africa. These are compounded by changes in land use/land cover, which in turn are driven mainly by economic and population growth, and urbanization. These factors create a feedback loop, which affects ecosystems and particularly ecosystem services, for example plant-insect interactions, and by consequence agricultural productivity. We studied effects of GEC at a local level, using a traditional coffee production area in greater Nairobi, Kenya. We chose coffee, the most valuable agricultural commodity worldwide, as it generates income for 100 million people, mainly in the developing world. Using the coffee berry borer, the most serious biotic threat to global coffee production, we show how environmental changes and different production systems (shaded and sun-grown coffee) can affect the crop. We combined detailed entomological assessments with historic climate records (from 1929–2011), and spatial and demographic data, to assess GEC's impact on coffee at a local scale. Additionally, we tested the utility of an adaptation strategy that is simple and easy to implement. Our results show that while interactions between CC and migration/urbanization, with its resultant landscape modifications, create a feedback loop whereby agroecosystems such as coffee are adversely affected, bio-diverse shaded coffee proved far more resilient and productive than coffee grown in monoculture, and was significantly less harmed by its insect pest. Thus, a relatively simple strategy such as shading coffee can tremendously improve resilience of agro-ecosystems, providing small-scale farmers in Africa with an easily implemented tool to safeguard their livelihoods in a changing climate.

## Introduction

The Intergovernmental Panel on Climate Change (IPCC) [1] predicts increases in the mean global temperature of up to 5.8°C by 2050, as well as more frequent ENSO (El Niño/La Niña) events, with climatic conditions expected to become generally more variable [1]. As a consequence of these global environmental changes (GEC) and increasing temperatures the life history traits of indigenous and invasive species may be impacted.

In addition to global warming caused by greenhouse gases, the effects of changes in land use/land cover on climate are an important part of GEC [2]–[4] which, unfortunately, are frequently overlooked [5]. For example, land use changes have been linked to alteration in surface energy and water balance [6], changes in land surface temperatures [7], [8] and habitat degradation and loss of biodiversity [9], [10]. As a result, modifications in local conditions may have an important impact on ecosystems and ecosystem services, for example plant-insect interactions, and ultimately on agricultural productivity.

GEC, including climate variability and changes in agricultural land use, will most likely have their severest effects on already vulnerable poor communities, particularly in the developing world [11], [12]. For instance, small-scale coffee farmers often rely directly on ecosystem goods and services for their subsistence, which make them vulnerable to change. Coffee (*Coffea arabica* L. and *C. canephora* Pierre ex A. Froehner) is produced mainly in the tropics and mostly by small-scale farmers on approximately 11 million hectares [13]. Environmental changes are already affecting many of these coffee growers, not only by directly influencing the coffee plants [14]–[17], but also indirectly by altering the population dynamics and incidence of coffee pests and diseases [18]–[20]. Thus, there is a need to better understand the interactions between agricultural intensification and GEC [21] to meet the challenge of developing resilient production systems for important agricultural commodities like coffee.

Increasing biodiversity in coffee plantations is a known and important strategy for building up the system's resilience e.g., [22]–[26]. Specifically, the practice of introducing shade trees into coffee plantations is considered a sound adaptation strategy to rising temperatures [25], [19]. Shade trees protect plants from microclimate variability [25], [26], from the effects of lower precipitation and reduced soil water availability, and reduce high solar radiation, hence buffering detrimental diurnal changes in air temperature and humidity [27]. In addition, coffee agroforestry has other positive effects on the crop like improved soil fertility, protection from insect pests [24], [28]–[30] and economic benefits for farmers [31]. Wild *C. arabica* grows as an understory tree of forests in East Africa [32], and until the 1970s it was predominantly cultivated under shade. However, due to increased market demand and the introduction of sun-resistant varieties, coffee growers had incentives for increasing productivity on their farms. This lead to a gradual elimination of shade trees on the plantations [13], [21]. These changes in production practices are becoming problematic in many coffee production areas due to higher pest and disease pressure, largely driven by GEC [33].

Using the coffee berry borer (*Hypothenemus hampei*), the most important coffee pest worldwide [34], [35], we document how environmental changes - particularly changes in temperature and rainfall – and coffee system type (shaded or sun-grown coffee) affect this major coffee pest. Starting in 1929 British entomologists investigated the eco-climates of coffee plantations in the Kiambu area of then colonial Kenya [36], [37]. Almost 100 years later we revisited the same coffee plots where those studies were conducted, to find out if and how changes in temperatures and land use pattern in the area have affected the cultivation of coffee in Kiambu.

## Results

### Climate parameters of the study area

Mean temperature in the Kiambu area has increased over the last century at a rate of 0.005°C per year from 1929 to 2009. However, sudden and conspicuous increases in temperature were noticed in 1950 and 1995 (Fig. S1), a tendency that accelerated particularly from 1991 onwards (Figs. S2 and S3). Precipitation data for Kiambu was available only from 1991 onwards (Fig. S4). Highest peaks in precipitation were recorded in the years 1997, 2006 and 2010, corresponding to the influence of El Niño events in East Africa (Fig. S5) [38].

### Land use change

**Figure 1:**
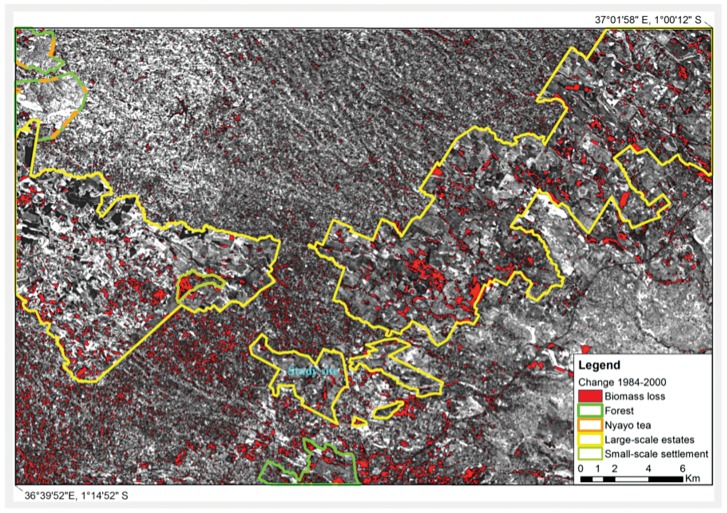
Land use change image 1984–2000 for the Kiambu area (Kenya) (background scene: Brightness image 1984).

**Figure 2:**
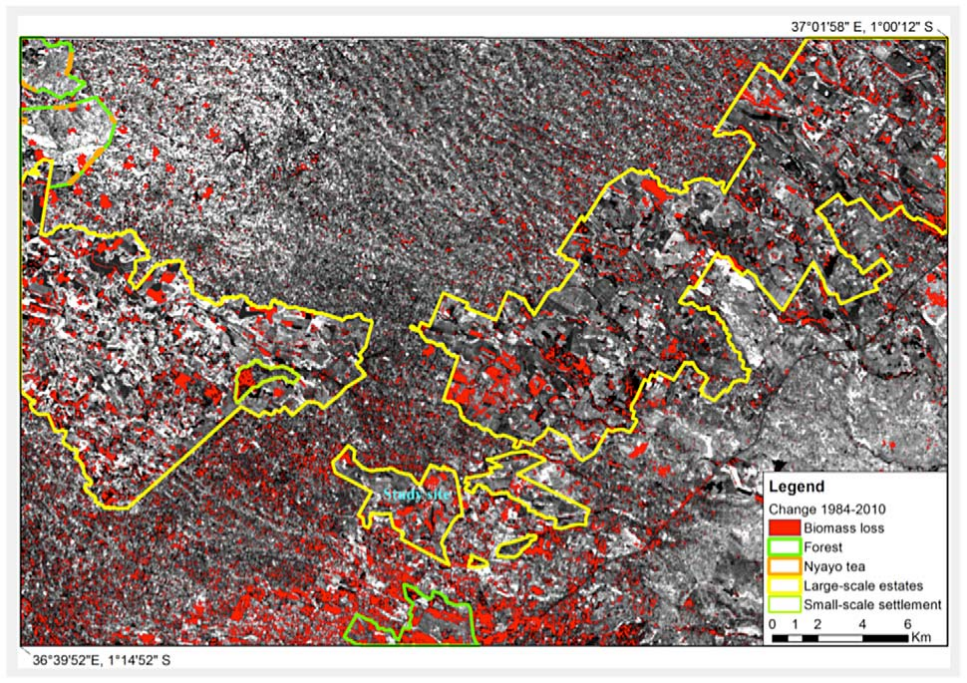
Land use change image 1984–2010 for the Kiambu area (Kenya) (background scene: Brightness image 1984).

The change image referring to the years 1984 and 2000 (Fig. 1) shows extensive changes in land use around the outskirts of Nairobi and more so in the large-scale farming belt, the relic of the “White Highlands”, compared to the change image of the years 1984 and 2010 (Fig. 2). Small-scale farming systems (middle-top in Fig. 1–2) show the least changes throughout the years. These land use and land cover (LULC) changes are governed by a combination of geographical, environmental and socio-economic factors. Population growth and economic development are the primary causes of these LULC changes. The proportion of Kenyans living in urban areas increased from 7.4% in 1960 to 21.3% in 2007 as a result of rural-urban migration [39].

### Plantation type and number of coffee berries

**Figure 3:**
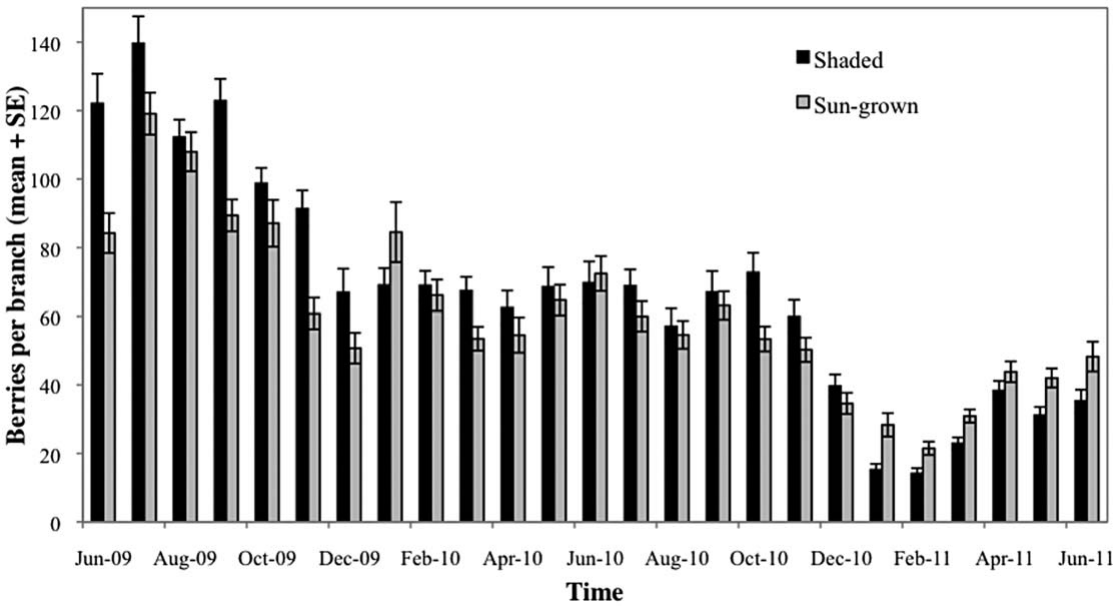
Mean number of berries per branch in a shaded and a sun-grown coffee plantation in Kiambu (Kenya) during the period June 2009 to June 2011.

The number of berries per branch varied significantly with plantation type (*F* = 13.86; df = 1, 178; *P* = 0.0003), sampling date (df = 24, 2712; *F* = 77.06; *P*<0.0001) and their interaction (*F* = 4.56, df = 24, 2712; *P*<0.0001). Overall, 10.8% more berries were recorded on shaded plantation compared to their sun-grown counterparts (LS means = 67.34 on shaded vis-à-vis 60.78 on non-shaded, Fig. 3). As shown in Fig. 3, significant fluctuations were observed in berry production per branch but with a general decreasing trend with time for both systems. Higher numbers of berries per branch were recorded in the shaded plantation during all the El Niño months except that or January 2010 (Fig. 3).

### Plantation type and *H. hampei* colonizing females

The distribution of colonizing *H. hampei* females in the different positions inside the berries varied with plantation type (log-likelihood test, *G* = 24.02, df = 3, *P* = <0.0001). In the sun-grown plantation, a higher proportion (44.5%) of *H. hampei* females was found inside the galleries at position 4 relative to the other three positions (χ^2^ = 1811.8, df = 3, *P* = <0.0001), whereas the proportion of females in position 2 (27.1%) and 3 (28.1%) were similar but significantly higher than position 1 (0.31%). In contrast, in the shaded plantation higher proportions of females were recorded in positions 2 (39%) and 3 (37.3%) (females attacking the exocarp only) (χ^2^ = 109.4, df = 3, *P* = <0.0001). In both planting conditions, the proportion of females found in position 1 was the lowest with 1% or less of females recorded.

Although the survivorship of *H. hampei* was not affected by the plantation type (*F* = 0.52; df = 1, 46; *P* = 0.472), significantly higher numbers of *H. hampei* females were recovered from the sun-grown plantation than the shaded one (*F* = 17.74; df = 1, 46; *P*<0.0001). The cumulative numbers of females retrieved from sun-grown berries was 18.2-fold higher than those recorded in berries collected from shaded trees (Shaded N = 1,622 individuals; Sun-grown N = 35,805 individuals). The proportion of coffee berries that were found with a hole in the exocarp (position 2) but where the colonizing female was absent was 31.86% (N = 94) in the shaded plantation and 24.12% (N = 1090) in the sun-grown plantation.

### Plantation type and *H. hampei* infestation level

**Figure 4:**
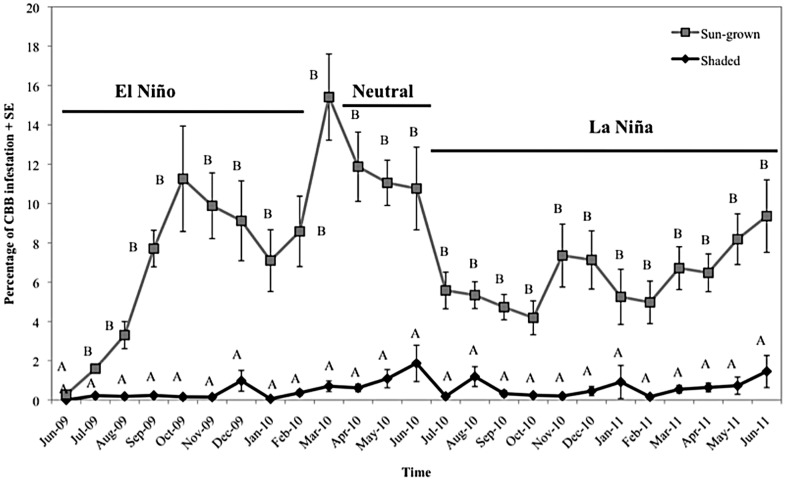
Mean *Hypothenemus hampei* infestation level in shaded and sun-grown coffee plantations in the Kiambu area (Kenya) during the period June 2009 to June 2011.

The percentage of berries infested by *H. hampei* differed significantly between plantation types (*F* = 370.51; df = 1, 2852; *P*<0 .0001), among sampling dates (*F* = 5.05; df = 24, 2852; *P*<0 .0001) and with their interaction (*F* = 4.40; df = 24, 2852; *P*<0 .0001). Significantly more berries were infested when trees were sun-grown (6.82%) compared to those grown under shade (0.55%), corresponding to ∼12-fold increase in berry infestation under full- sunlight growing conditions (Fig. 4). Monthly *H. hampei* infestation levels in the sun-grown plantation fluctuated between 0 and 16%, exceeding the 5% coffee berry borer economic threshold for all months except June and July 2009 (Fig. 4). In contrast, the infestation level observed for shade-grown berries did not exceed 2% at any time.

### Temperature and rainfall and *H. hampei* infestation and survival

**Figure 5:**
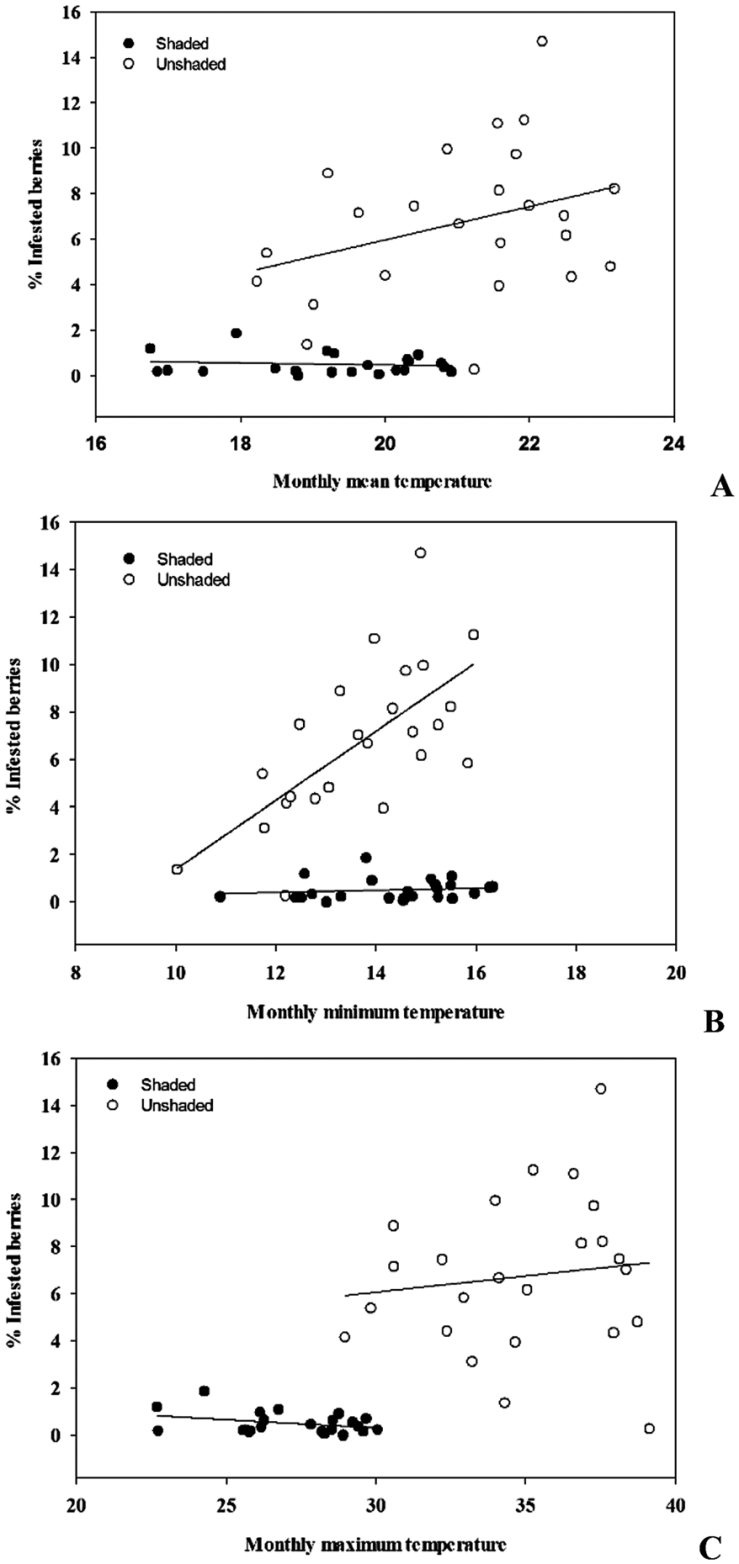
Effect of mean, maximum and minimum monthly temperature on *Hypothenemus hampei* infestation level under shaded and sun-grown coffee plantations in Kiambu (Kenya) between June 2009 and June 2011.

**Figure 6:**
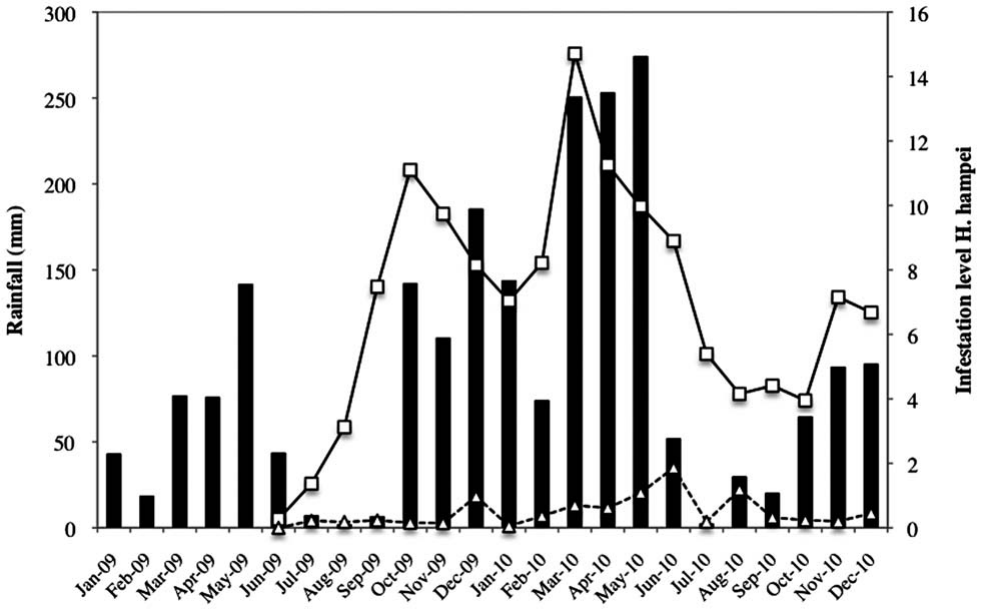
Effect of rainfall (mm) on *Hypothenemus hampei* infestation level under shaded and sun-grown coffee plantations in Kiambu (Kenya) between June 2009 and June 2011.

Shading coffee trees significantly changed the microclimate of the plantation (Fig. S6). Monthly temperatures increased with time in both planting conditions, which is consistent with historical trend observed from 1929 to 2011 (Fig. S1, S2 and S3). Paired t-tests revealed that the averages of mean (*t* = 24.53, df = 66, P<0.0001) and maximum (*t* = 33.02, df = 66, P<0.0001) temperatures were significantly higher in the sun-grown plantation relative to the shaded one. In contrast, the sun-grown plantation had significantly lower minimum temperatures (*t* = 13.14, df = 66, *P*<0.0001). Higher mean and maximum temperatures in the sun-grown plantation were associated with increased borer infestation levels compared with those seen in the shaded plantation (Fig. 5A, 5C). The strongest positive effect on infestation was recorded for minimum temperature in the sun-grown but not in the shaded system (Fig. 5B). Parallel line analysis revealed that infestation level was positively correlated with increasing maximum temperature in the sun-grown plantation but slightly negatively affected in the shaded plantation. Likewise, no effect of mean temperature on *H. hampei* infestation was recorded in the shaded plantation. Although *H. hampei* infestation level remained unchanged in the shaded system with minimum temperature, the effect of rising minimum temperature significantly increased infestation in the sun-grown plantation. As with temperature, the effect of rainfall on *H. hampei* infestation of coffee berries was only significant under the sun-grown system (Fig. 6). Infestation level (IL) increased with monthly total rainfall (IL = 4.0+0.03*Rainfall, t = 4.84, df = 17, P = 0.0002, R^2^ = 0.58) in the sun-grown trees, while rainfall did not significantly influence *H. hampei* infestation in shaded trees (IL = 0.3+0.002*Rainfall, t = 1.15, df = 17, P = 0.27, R^2^ = 0.07).

## Discussion

An important consequence of global climate change is human migration [40]. Climate change with its induced variability in rainfall pattern, rise in temperature and higher prevalence of extreme weather events, is predicted to have particularly serious impacts on agriculture [1]. Therefore, countries whose economies depend heavily on agriculture for their development, including most of sub-Saharan Africa, may be hardest hit by a change in climate [12]. Climate change consequently leads to internal displacement of people, and is hence a new key determinant of urbanization [41]. At the same time, urbanization is an example of how land use change modifies regional climate [6]. This interaction between global climate change, human migration/urbanization, economic development and the inherent modification of the landscape and, as a consequence, of the regional climate create a feedback loop where ecosystems and people may be severely affected.

In sub-Saharan Africa, Kenya and its capital Nairobi are an example of such a loop. The population in the country has noticeably grown during the last century from 2.5 million inhabitants in 1897 to 40 million in 2010 [42]. Since the latter part of the 20^th^ century the population of Kenya, and in particular that of Nairobi, has gone up sharply; that of Kenya from 15.3 million in 1979 to 40 million in 2010, and that of Nairobi from 827,775 in 1979 to 3.1 million in 2009. [43]. The Kiambu area in the outskirts of Nairobi has been traditionally and for many decades, one of the most important coffee production areas of the country. A consequence of this accelerated population growth and urbanization process of the last decades, has been the transformed landscape of Kiambu. The sharply increasing human population densities of Kiambu - 194 people/km^2^ in 1969 to 638 people/km^2^ in 2011 [44], [45] – are fueling the pressure on the land. Recently, coffee production has started to be replaced by upstream market real state developments [46], [47] and the few coffee farms that remain have responded by increasing management intensification in order to maintain their productivity.

Our analyses of land use change in Kiambu confirm the dramatic transformation since 1984, with the most noticeable changes in vegetation having occurred in areas around the larger coffee plantations. These plantations were originally pre-colonial “white settlements” and coffee estates. Before 1933, Africans were not allowed to grow coffee in Kenya, and it was only after 1948 that the colonial authorities granted them permission to grow coffee in areas other than Kisii, Embu or Meru [48]. Kiambu's large coffee estates were and still are characterized by an intensified cultivation scheme under very low or no shade (Fig. 2). On the other hand, small-scale diverse production systems that include not only coffee but also maize, beans, timber and fruit trees (upper middle section of Figs. 1–2) show little vegetation change over time, which can be attributed to normal fluctuations in vegetation index or abandonment of coffee farms, indicating high resilience in the system. The small-scale farms around the original pre-colonial “white settlements” usually belong to numerous African families that with time have divided their land into very small units (of max. 1–2 acres each) where every member of the family (siblings) cultivates coffee and food crops in a diversified manner.

The striking changes in land use as a consequence of urbanization have had drastic effects on the prevalent temperature conditions of the Kiambu area. Our analysis of 82 years (1929–2011) of location specific climate data indicates an increase in temperature at a rate of 0.005°C per year, matching the IPCC estimates for Africa [1]. It is noteworthy to mention that temperature recordings for the study area were gathered from weather stations within the areas dominated by large-scale coffee estates or directly at the farm where the field study was conducted.

Unfortunately, we lack detailed high-resolution satellite images or aerial photographs for the Kiambu area between 1950–1960s when a sudden increase in temperature in the area was recorded (Fig. S1), but most probably this rise in temperature coincided with a series of strong La Niña events that took place during those years [49]. During the same period, accounts by British entomologists link changes in the environmental conditions with a coffee berry borer outbreak in coffee plantations in Kiambu: “Recent inspections have shown that the coffee berry borer beetle is present in great numbers on certain estates in Kiambu […] the reason for its recent increase is not clear [but] may be a cyclical event correlated with a climatic change” [50].

Ecosystems are influenced by the dynamic interactions between climatic factors, plants, pests, their natural enemies and the surrounding ecosystem including humans. GEC together with changes in land use [51] influence population dynamics at all trophic levels [52]–[55]. In agricultural systems, particularly that of coffee, herbivorous insects can have significant impacts on plant productivity and can become a constant problem for farmers [26]. Pioneering work on the effects of eco-climatic conditions on insect pests in shaded and sun-grown coffee, was carried out by Kirkpatrick in the 1930s [36], [56], in the same coffee plantation in Kiambu where our field study was conducted. Almost one hundred years later, we decided to come back to the same coffee plantation to find out if and how things have changed.

We were interested in studying how coffee is affected by changing environmental conditions via the indirect effects of a herbivore. We used the coffee berry borer because of its economic importance [34], [35] and because problems with agricultural insect pests are forecasted to intensify in the future [57]–[59].

In order to simulate contrasting microclimatic conditions and management intensification levels and their effects on the coffee plant and the pest, two different coffee systems – shaded and sun-grown plantations – were compared, with the objective of investigating whether shaded coffee is indeed more resilient to climatic variability than sun-grown and to evaluate the potential use of shade trees as an adaptation strategy to changing environmental conditions.

During the course of the study, mean temperatures in the sun-grown plantation were roughly 2°C higher than in the shaded one. Minimum temperatures were higher in the shaded system, on the other hand, indicating that the shaded system was less prone to drastic temperature fluctuations. Our findings are in line with previous research suggesting that shade trees change the microclimate of the coffee plantation and mitigate microclimatic extremes [36], [25], [60]–[61].

According to the reported thermal tolerance of coffee berry borer [19], the temperatures we recorded imply that *H. hampei* could develop in both plantation types, but that borer development would be much faster in the sun-grown system. Jaramillo et al. [19] calculated that for every 1°C rise in the thermal optimum, the maximum intrinsic rate of increase of the pest would increase by an average of 8.5%. Consequently pest populations in the sun-grown plantation would rise 17% more than in the shaded one. We recorded a 12.4-fold increase in berry infestation and a 18.2-fold increase in the cumulative number of female beetles in the sun-grown versus the shaded plantation, confirming results of Jaramillo et al. [19] model as well as corroborating observations made in coffee plantations in Mexico [62]. Finally, pest infestation levels in the sun-grown plantation exceeded the 5% economic threshold on nearly all sampling dates, whereas in the shaded plantation this threshold was never reached. We also noticed a marked influence of rainfall pattern on the *H. hampei* infestation level but interestingly only under sun-grown conditions (Fig. 6). Rainfall triggers colonization flights of *H. hampei* females [63]–[64]; an effect that is enhanced by high temperatures in the plantation [64].

Not only infestation of but also damage to the berries was more significant in sun-grown coffee. Here, colonizing females were more frequently found inside the berries constructing galleries and ovipositing, while females in the shaded plantation were more often found in the exocarp. Additionally, considerably more berries with a hole in the exocarp but without the insect were found in the shaded plantation, implying that the colonizing females probed the berries but did not find suitable conditions for gallery construction and egg laying. Delayed development and maturation of berries under shade and consequent changes in their final biochemical composition may explain this finding [27], [65], as well as changes in the emission of host location olfactory clues used by the colonizing females [66]. Additionally, we found that the exocarp of coffee berries grown under shade was significantly thicker than those from sun-grown coffee (shade 4.74 mm; sun-grown 4.37; F = 49.29, *P*<0.0001).

Finally coffee trees growing under shade had 10.8% more berries per branch compared to the sun grown trees. During the course of this study, Kenya experienced the influence of both ENSO (El Niño/La Niña) and Neutral (normal) conditions. These events are accompanied by marked changes in rainfall in the area as seasonality in East African rainfall is controlled primarily by the Intertropical Convergence Zone (ITCZ), which is driven to a large extent by ENSO [38]. Higher numbers of berries per branch were recorded in the shaded plantation during almost all the months with El Niño or Neutral conditions (Fig. 3), most likely matching a period of higher water availability in the plantation. Shade improves the water status of the soil because of reduced evapotranspiration in the agro-ecosystem and an increased ground cover (mulch) and decreased abundance of weeds [61].

In this study we combined entomological assessment of a key coffee pest with 82 years of climate data, as well as spatial and demographic data, to assess the impact of GEC on the economically most important agricultural commodity and to test the utility of an adaptation strategy that is easy to implement, hence suitable for the millions of small-scale coffee growers in the developing world. Our study illustrates the remarkable changes in human population density, vegetation cover and land-use, local climate and the interconnections of all these factors in the peri-urban environment of an East African capital over nearly 100 years. Our study not only demonstrates the urgent need to study climate-change at regional spatial scales, but also the importance of local factors. Moreover, we were able to illustrate how these effects can affect agricultural productivity, mainly through their impacts on higher trophic levels like insect herbivores. However, we also showed that a relatively simple strategy, the introduction of shade trees in coffee plantations, could markedly improve the resilience of an agroecosystem, providing small-scale farmers in Africa with a much-needed, easy to adopt, tool to safeguard their livelihoods in a changing climate.

## Materials and Methods

### Study site

The study was conducted in a commercial coffee plantation in Kiambu district (Central province), Kenya (Fig. 2 and Fig. S7). No specific permits were required for the described field studies. Two plots of *Coffea arabica* var. Ruiru 11 (planting density 1.82×1.82 m) were selected; a shaded plot (65% canopy cover) (1°11′27.15″S; 36°49′23.03″E. altitude 1,722 m.a.s.l) with 300 coffee trees and 15 shade trees (two avocado (*Persea Americana* L.), 1 mango (*Mangifera indica* L.) and 12 grevillea (*Grevillea robusta* (A. Cunn.)), and a sun-grown plot (10% canopy cover from bananas at one edge of the plot) (1°11′24.22″S; 36°49′25.10″E. altitude 1,720 m.a.s.l) with 280 coffee trees. Canopy cover (% shade) was estimated visually four times during the course of the study, both during the rainy and dry season according to Teodoro et al. [67]. Trees in both plantations were planted in January 1999, and both plantations were under the same agronomic management. No *H. hampei* control measures were used in either plot during the course of the study.

### Data collection

Data on *H. hampei* infestation level were collected every two weeks from June 2009 to June 2011. In both the shaded and the sun-grown plots, 15 trees were randomly chosen at each evaluation date. To assess *H. hampei* infestation level, two branches per tree were selected. There, total number of berries and total number *H. hampei* infested berries were counted. At each evaluation date, all the infested berries from both plots were individually collected and taken to the International Center of Insect Physiology and Ecology (*icipe*) laboratories in Nairobi, Kenya, for dissection. In the laboratory, numbers of live, dead and absent *H. hampei* colonizing females (i.e., berries that had a penetration whole in the exocarp but where the insect was not present), the position of colonizing females inside the berries (see below) and number of coffee berry borer life stages (i.e., eggs, larvae, prepupae, pupae and adults) were assessed at each evaluation date. Four different positions based on the insect location within the berry were identified: position 1, colonizing female starting to colonize a new berry but the penetration in the exocarp has not taken place; Position 2, colonizing female has bored a whole into the exocarp but has not yet reached the endosperm; Position 3, colonizing female has started to bore into the endosperm but not to oviposit; Position 4, colonizing female had constructed one or more galleries in the endosperm, and eggs or other immature stages are found inside the galleries.

### Climate data

To assess the temperature in the plots, ten data loggers (HOBO U12 J, K, S, T Thermocouple data logger, Onset Computer Corporation MA, USA) were installed in each plantation type (shaded or sun-grown) in June 2009. Temperature was recorded every half hour for the whole study period. The loggers were hung at 150 cm from the ground. In order to accurately characterize the temperature of the plots, the data loggers were place in three different locations within the plots: within the trees in the area of highest concentration of berries, in the rows between the trees, and at the edge of the plantation. In the case of the shaded plots, we additionally placed them in areas of highest, medium and low shade concentration.

In addition, historic climatic data for the farm and for the Kiambu area (Tmax, Tmin, Tmean, and precipitation) were gathered from the studies on eco-climate of coffee plantation by Kirpatrick [36] and from McDonald [37], and provided by the Kenya Meteorological department.

### Land use change analysis

Kiambu area is located north of Nairobi, Kenya, in UM 2–3 agro-ecological zones (between latitude 1°14′52″ to 1°00′12″S and longitude 36°39′52″ to 37°01′58″E) (Fig. S7). It covers a total area of 103 km^2^ characterized by warm sub-humid climate with annual rainfall ranging from 900 to 1400 mm. The satellite data used in this analysis came from the Landsat-TM (path: 168, row: 61) on December 17, 1984 and on August 19, 2010, and the Landsat-ETM (path: 168, row: 61) on February 21, 2000 (Table 1). The images were selected with as little cloud cover as possible, and were chosen at a time for better characterization of vegetation. Additionally, topographic maps, Africover land cover data, and SRTM 90 m digital elevation data for study area were used. To create a multi-temporary remote sensed data set for change detection, all images were geometrically corrected to the Universal Transverse Mercator coordinate system (zone 37) and then radiometrically normalized. ETM+ image were rectified to the same geo-referencing system as TM image using a geometric polynomial transformation model (first degree) and nearest neighbor resampling method. The total RMS error is around 0.5 pixels, which is suggested as acceptable for change detection.

**Table 1:** Properties of the satellite data used in the study.

Satellite	Acquisition date	Spectral bands	Ground resolution (m)
Landsat TM	17-12-1984	1–7	30
Landsat ETM+	21-02-2000	1–7	30
Landsat TM	19-08-2010	1–9	30

TM, thematic mapper; ETM+, Enhanced Thematic Mapper Plus.

#### Change Vector Analysis

Change Vector Analysis technique was applied to multi-temporal data to compare the differences in the time-trajectory of the tasseled cap greenness and brightness for two successive time periods – 1984/2000 and 2000/2010. The tasseled cap was selected as biophysical indicator [68]. The magnitude of vectors was calculated from the Euclidean distance between the difference in positions of the same pixel from different data-takes within the space generated by the axes greenness and brightness, as follows (Eq. 1):
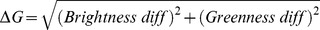
Where *ΔG* includes all the change information between the two dates for a given pixel. The angle of the vectors, which indicates the type of change that occurred, varies according to the number of components used. Only components Greenness and Brightness were used in this study, thus, only four classes of change were possible: no change, low biomass loss, high biomass loss and water. A threshold of final magnitude was defined for each one of the change classes through an interactive adjustment.

### Statistical analysis

A repeated measures analysis was used to analyze the differences in the number of berries per branch, the number of immature stages, the total number of females per berry, the total number of eggs per female and in the percentage of berries infested by *H. hampei*, between shaded and sun-grown plots using the PROC MIXED of SAS. Where significant effects were obtained for the growing condition, least squared means of treatments were separated using Tukey's HSD test. To stabilize variances, count data were log (x+1)-transformed and percentages were arcsine-transformed before analysis, but untransformed means are presented in tables and figures. Position and survivorship of colonizing *H. hampei* females were compared across observation dates using log-likelihood ratio test (G-test). To determine the effects of temperature on *H. hampei* infestation level, monthly mean values were correlated with average monthly minimum, maximum and mean temperatures for each plantation type. Subsequently, a covariance analysis using PROC MIXED of SAS was run to determine the joint impact of monthly temperatures and plantation type and their interaction on berry infestation level by *H. hampei*. Parallel line analysis compared the regression lines of both types of plantation [69].

## Supporting Information

Figure S1 **Mean temperature in Kiambu area (Kenya) during the period 1929–2011.**
(TIF)

Figure S2 **Maximum temperature recorded in Kiambu (Kenya) from 1992 to 2011.**
(TIF)

Figure S3 **Minimum temperature recorded in Kiambu (Kenya) from 1992 to 2011.**
(TIF)

Figure S4 **Precipitation (mm) in Kiambu (Kenya) from 1991 to 2010.**
(TIF)

Figure S5 **Relationship between the Oceanic Nino Index (ONI) and precipitation in Kiambu, Kenya, during the period 1991–2011.**
(TIF)

Figure S6 **Temperature recorded in Shaded (SH) and Sun-grown (USH) coffee plantations in Kiambu (Kenya) during the study period (June 2009–June 2011).**
(TIF)

Figure S7 **Study site.**
(TIF)
