# Supplementary figures and images for: Climate Change or Urbanization? Impacts on a Traditional Coffee Production System in East Africa over the Last 80 Years
Source: PLoS One. 2013 Jan 14;8(1):e51815. doi: 10.1371/journal.pone.0051815 (PMC3544928; doi:10.1371/journal.pone.0051815)

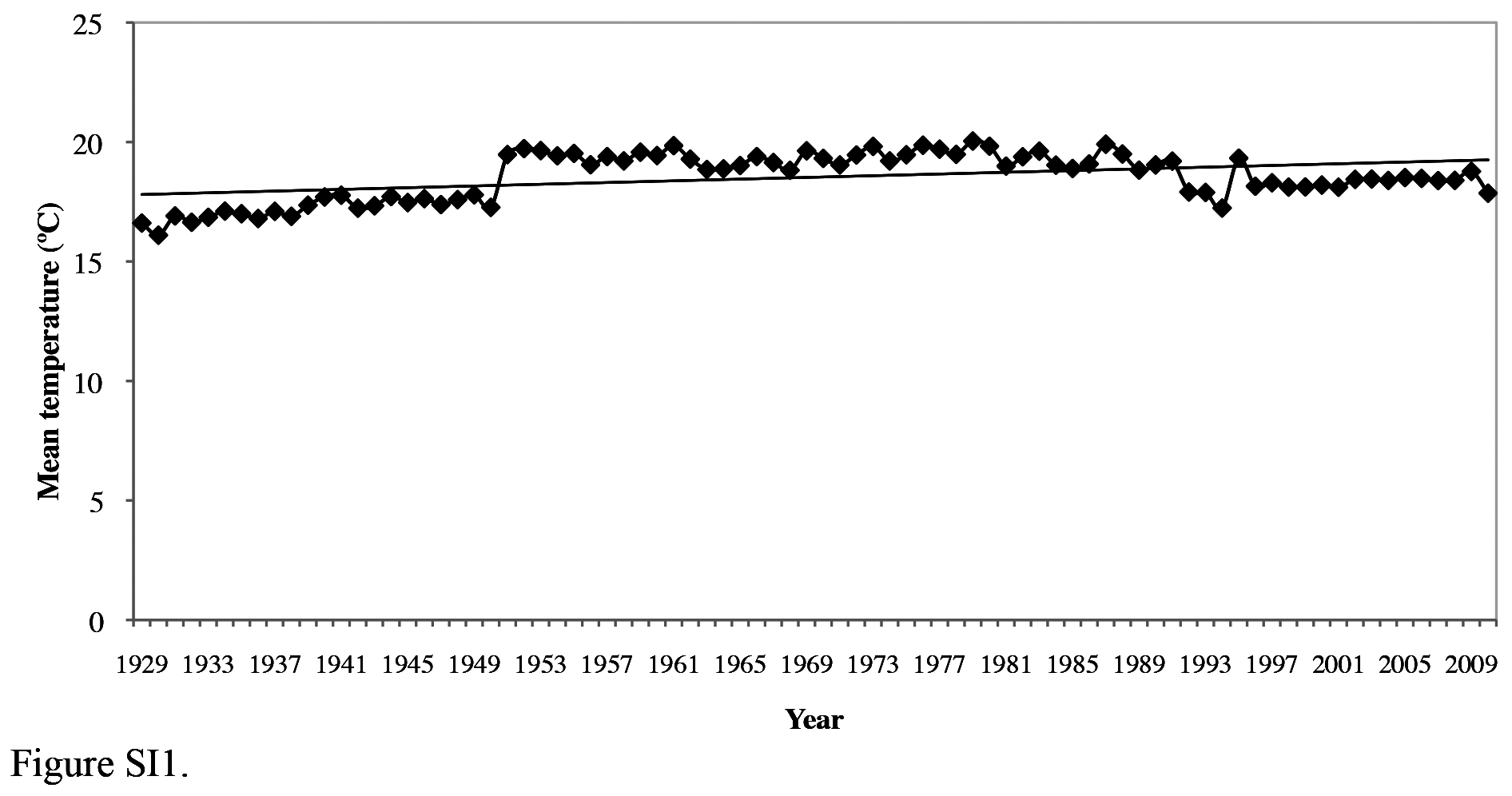

Supplement: Figure S1 — Mean temperature in Kiambu area (Kenya) during the period 1929–2011. (TIF) [file pone.0051815.s001.tif]

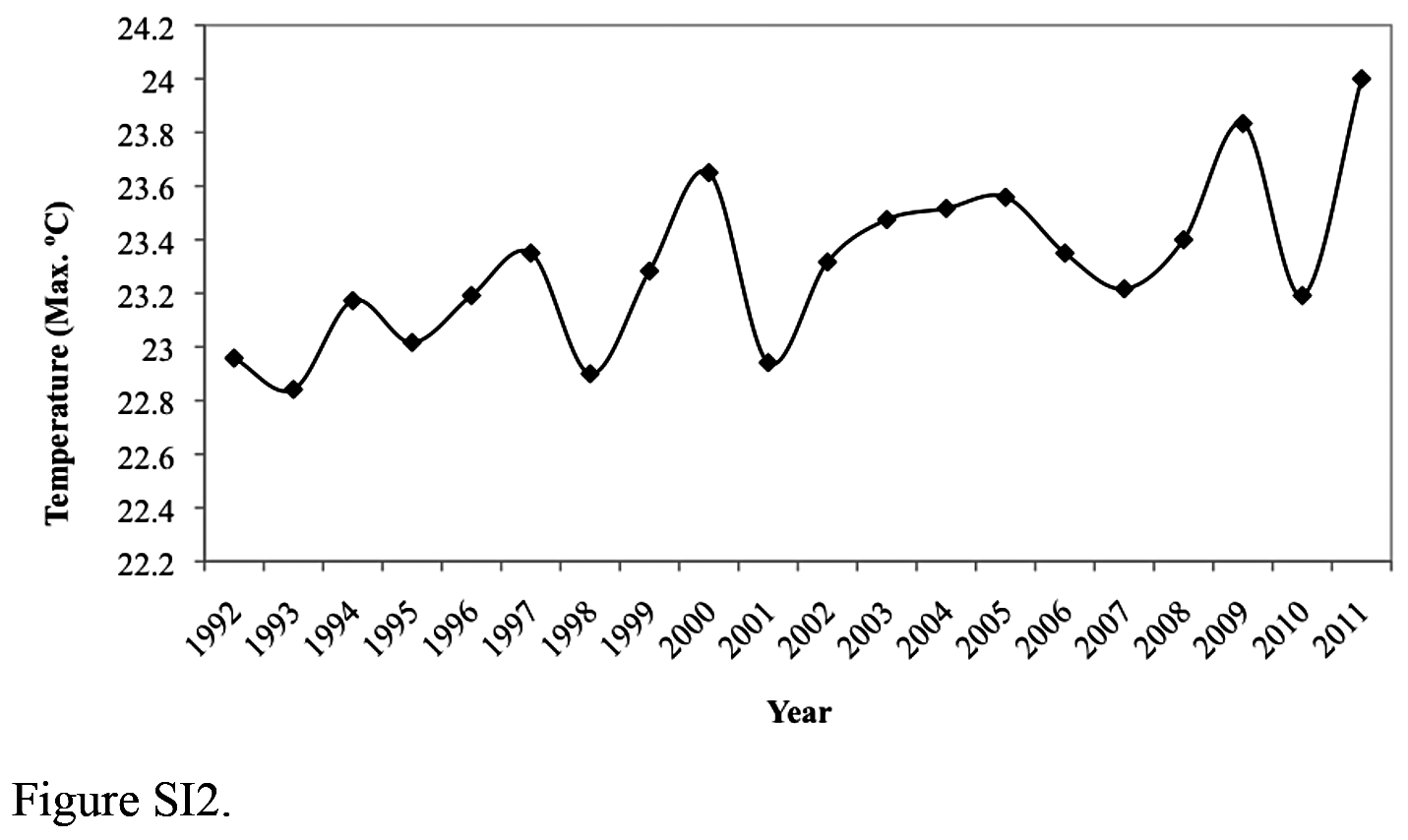

Supplement: Figure S2 — Maximum temperature recorded in Kiambu (Kenya) from 1992 to 2011. (TIF) [file pone.0051815.s002.tif]

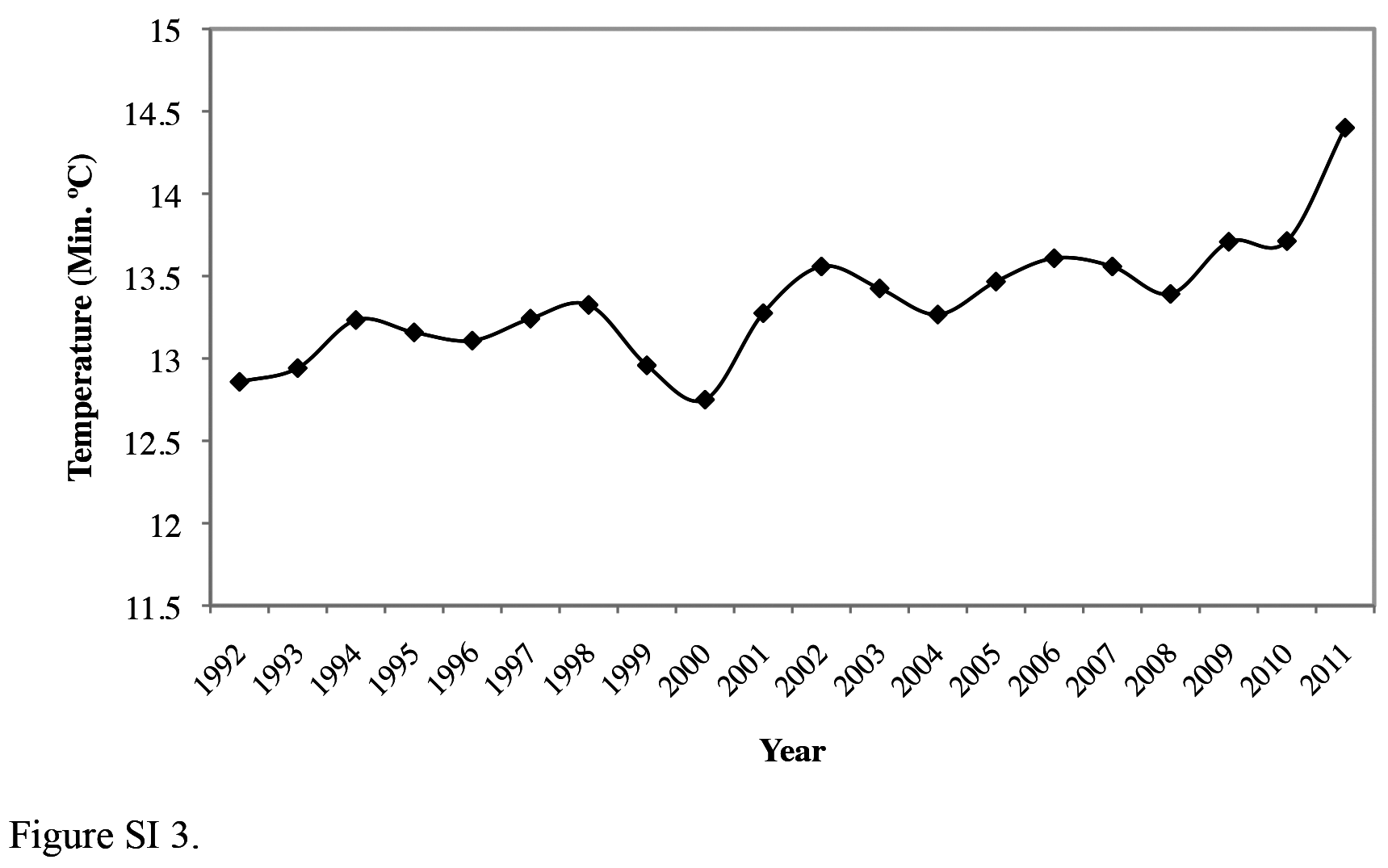

Supplement: Figure S3 — Minimum temperature recorded in Kiambu (Kenya) from 1992 to 2011. (TIF) [file pone.0051815.s003.tif]

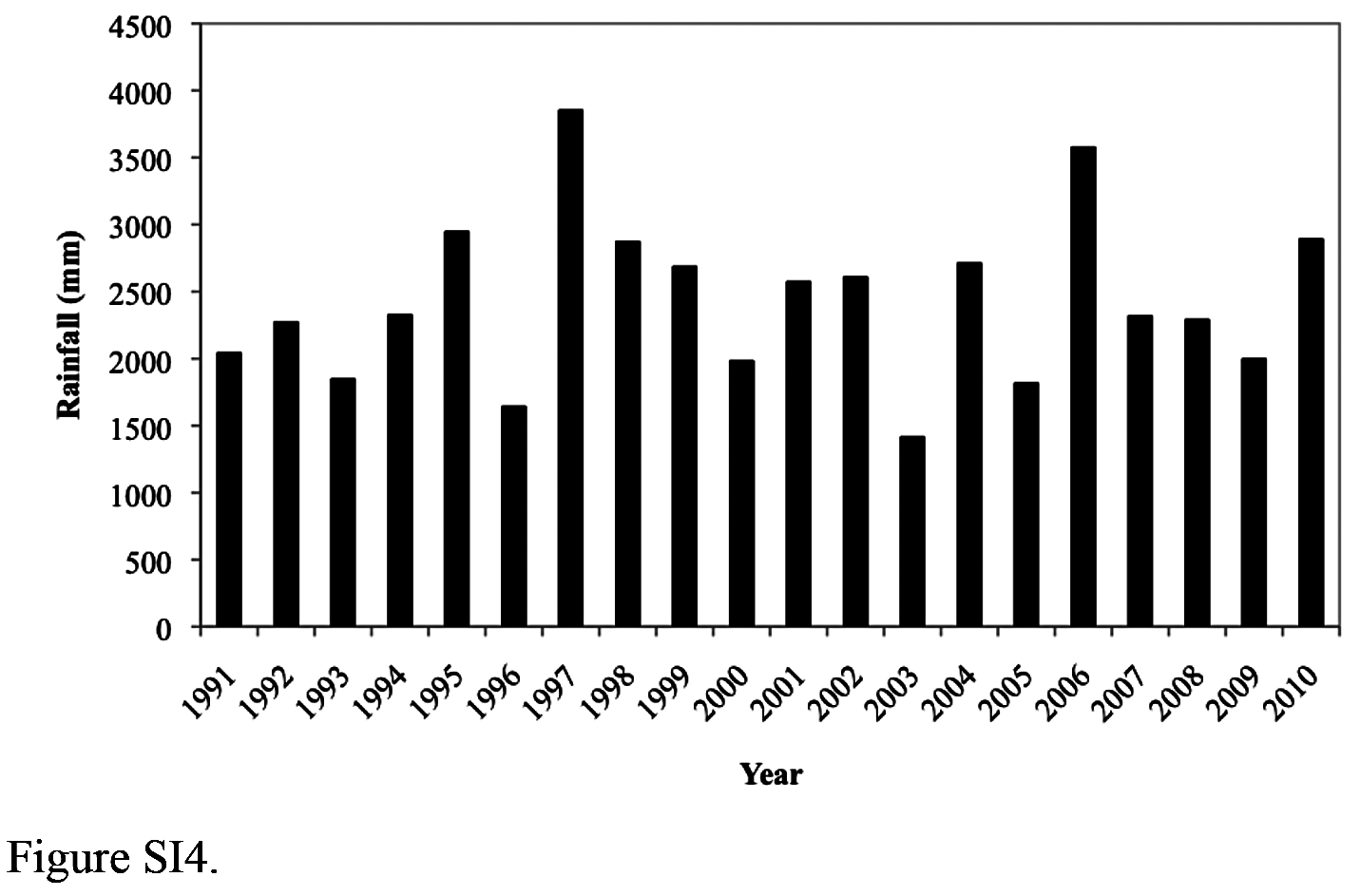

Supplement: Figure S4 — Precipitation (mm) in Kiambu (Kenya) from 1991 to 2010. (TIF) [file pone.0051815.s004.tif]

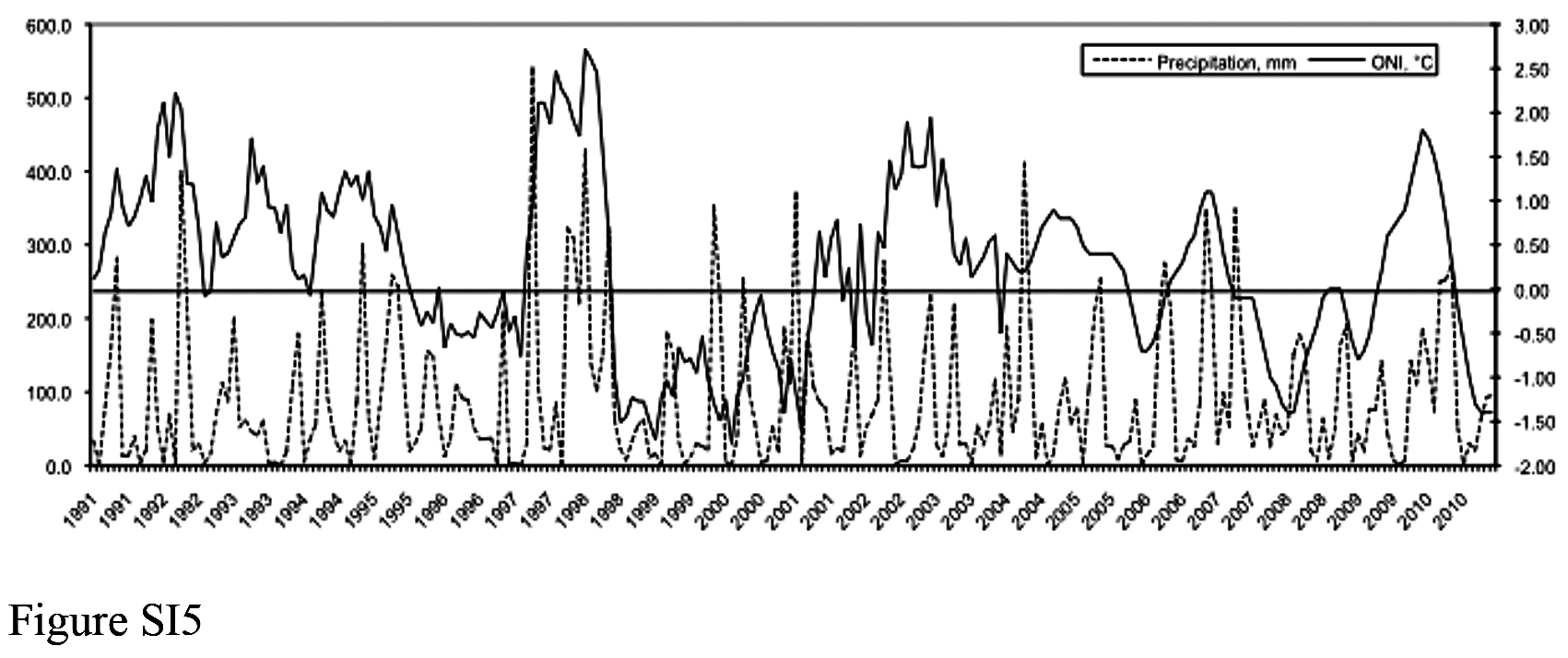

Supplement: Figure S5 — Relationship between the Oceanic Nino Index (ONI) and precipitation in Kiambu, Kenya, during the period 1991–2011. (TIF) [file pone.0051815.s005.tif]

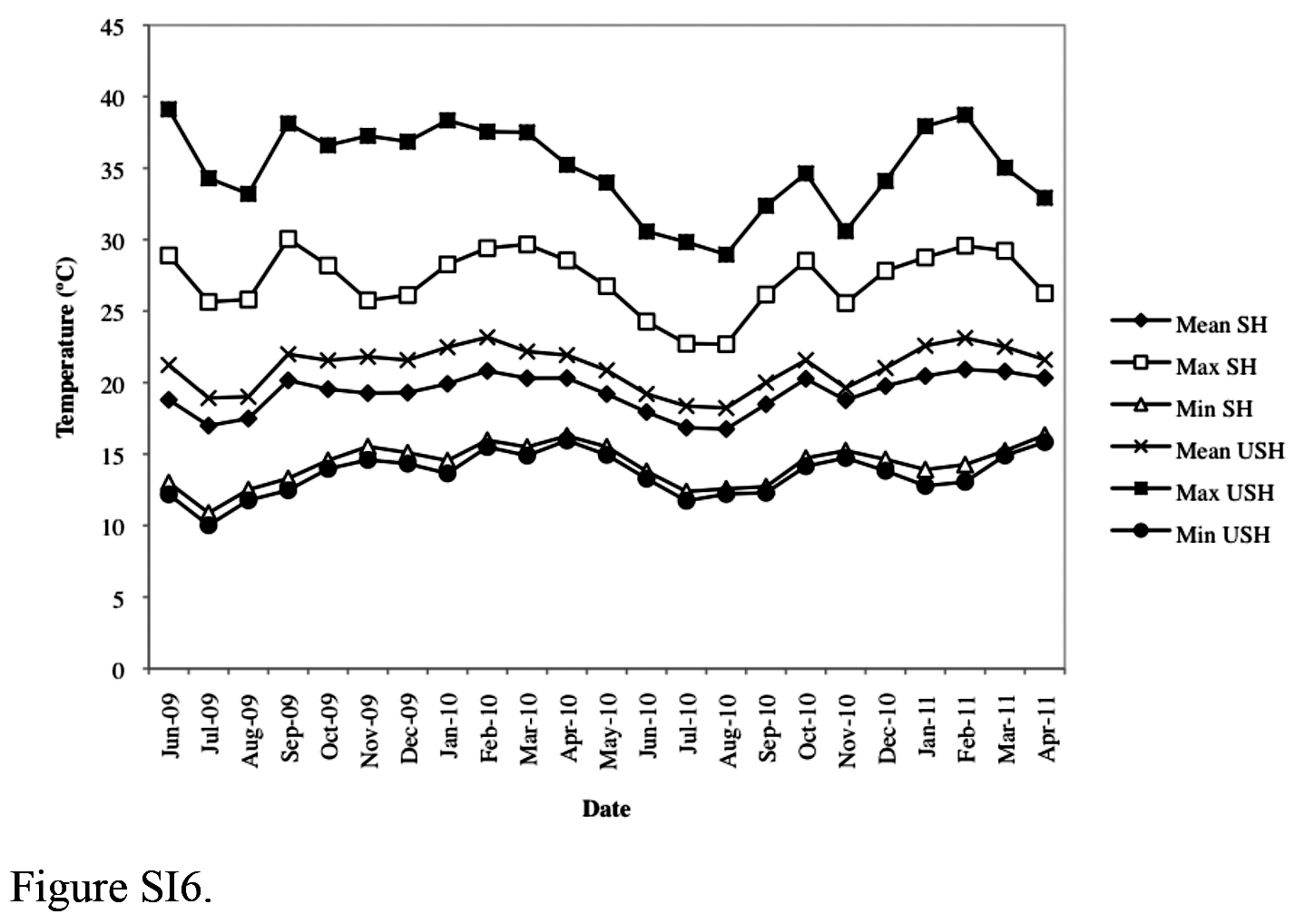

Supplement: Figure S6 — Temperature recorded in Shaded (SH) and Sun-grown (USH) coffee plantations in Kiambu (Kenya) during the study period (June 2009–June 2011). (TIF) [file pone.0051815.s006.tif]

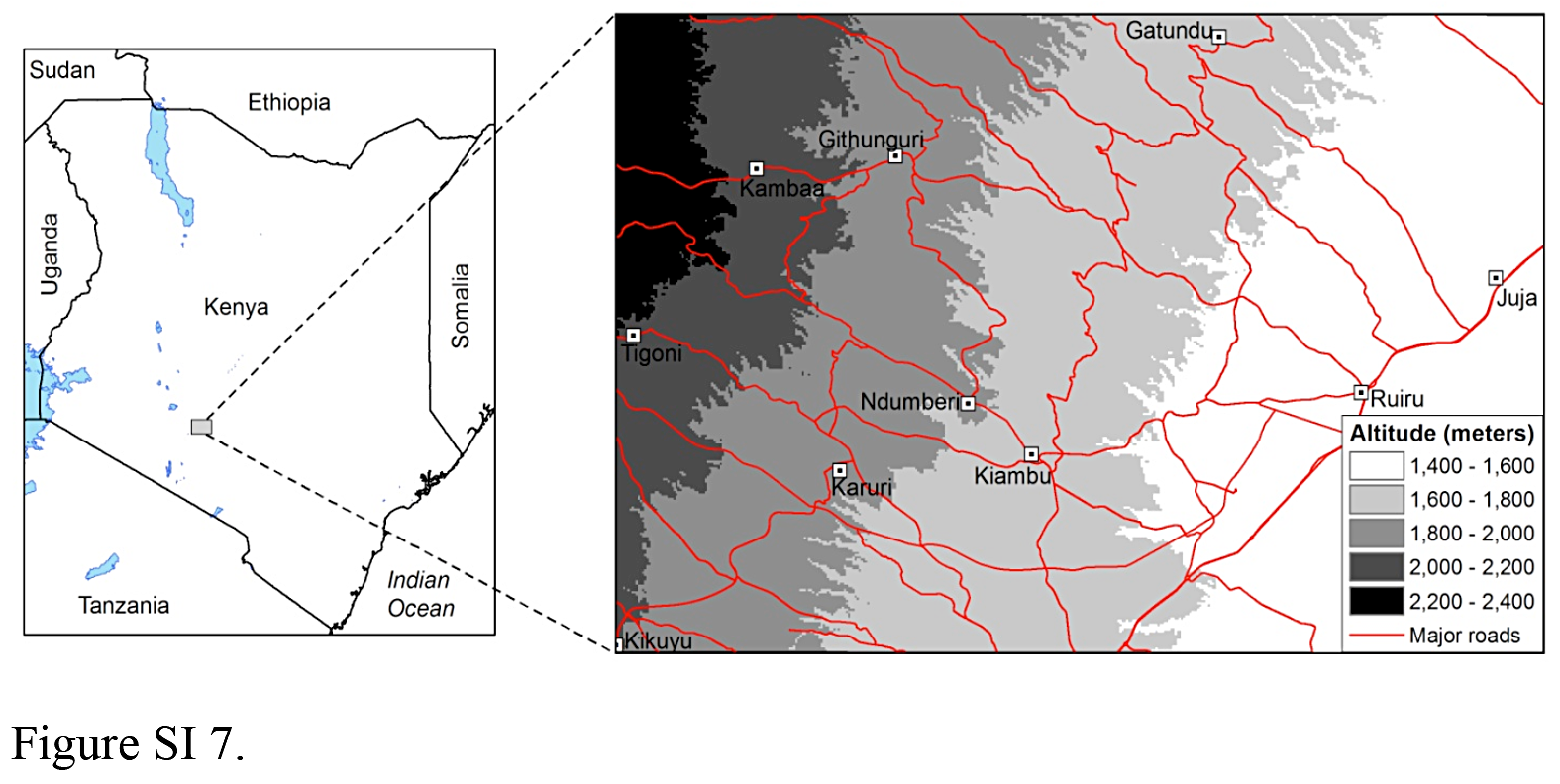

Supplement: Figure S7 — Study site. (TIF) [file pone.0051815.s007.tif]
